# BrainBike peptidomimetic enables efficient transport of proteins across brain endothelium†

**DOI:** 10.1039/d3cb00194f

**Published:** 2023-12-11

**Authors:** Maria C. Lucana, Roberta Lucchi, Fabien Gosselet, Cristina Díaz-Perlas, Benjamí Oller-Salvia

**Affiliations:** a Institut Químic de Sarrià (IQS), Universitat Ramon Llull Barcelona 08017 Spain benjami.oller@iqs.url.edu cristina.diaz@iqs.url.edu; b Université d’Artois, Blood-Brain Barrier Laboratory Lens 62300 France

## Abstract

Protein therapeutics cannot reach the brain in sufficient amounts because of their low permeability across the blood–brain barrier. Here we report a new family of bicyclic peptide shuttles, BrainBikes, capable of increasing transport of proteins, including antibody derivatives, in a human cell-based model of the blood–brain barrier.

## Abstract 2

BrainBikes, a new family of bicyclic brain shuttle peptides, can efficiently transport protein therapeutics across brain endothelium.

## Graphical abstract

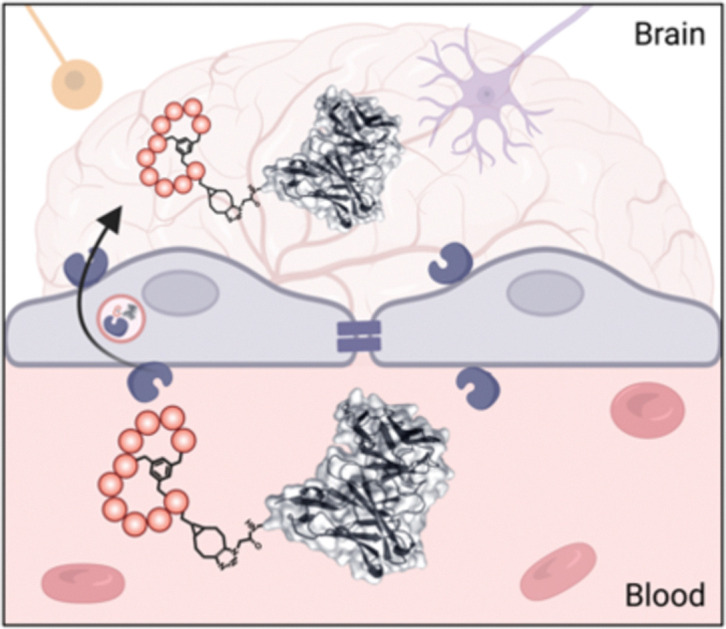

The blood–brain barrier (BBB) is a formidable obstacle that restricts access of most small drugs and all large therapeutics to the brain.^1^ However, endogenous transport mechanisms at the BBB can be hijacked using brain shuttles to enhance the transport of drugs. Receptor-mediated transcytosis (RMT) has been shown to be the most effective non-invasive pathway for the transport of large therapeutics across the BBB.^2^ Although many molecules have been developed as brain shuttles over the past three decades capitalizing on RMT, transport efficiency is still inadequate. Moreover, transport capacity of each shuttle is highly dependent on the cargo to be transported.^3^ Therefore, there is a need to expand the brain delivery toolset by developing new shuttles with distinct properties.

Peptide brain shuttles are easy to produce, characterize, and conjugate when compared to larger proteins such as antibodies. However, linear peptides with proteinogenic amino acids have low resistance to proteases. We and others have previously shown that increasing resistance of peptide shuttles to proteolysis greatly enhances their transport capacity (Fig. 1).^4–6^ We initially utilized a retro-enantio or retro-inverso approach, which enables very high protease resistance by replacing all amino acid residues by their D-counterparts in the reversed order.^6^ However, this strategy often results into a decrease in affinity, which may impact transport capacity. By contrast, cyclic peptides from natural sources such as venoms have an intrinsically high resistance to proteases.^7,8^ Moreover, cyclic peptides may provide enhanced affinity over their linear counterparts due to decreased binding entropy.^9^ Cysteine-rich natural peptides are complex to synthesize and we have shown that minimized monocyclic versions of neurotropic peptide toxins, such as MiniAp-4 and MiniCTX3, can be developed as efficient brain shuttles.^10,11^ However, monocyclization may provide high resistance to proteases and affinity only in very short peptides. In the present work, we propose a strategy that could be applied to a wider variety of peptide shuttles, which generally range from 7 to 12 amino acids.^3^ To this end, we proposed generating bicyclic shuttles from linear parent peptides. An elegant approach to produce isomerically-defined bicyclic peptides with exquisite regioselectivity is by reacting three residues, for instance cysteines, with a trifunctional linker.^12^ Such bicyclic peptides have been extensively used as protease inhibitors, high-affinity binders, and tumor-targeting ligands.^13–16^ However, this type of scaffold has not been applied to generate shuttles to transport cargoes across the BBB.

**Fig. 1:**
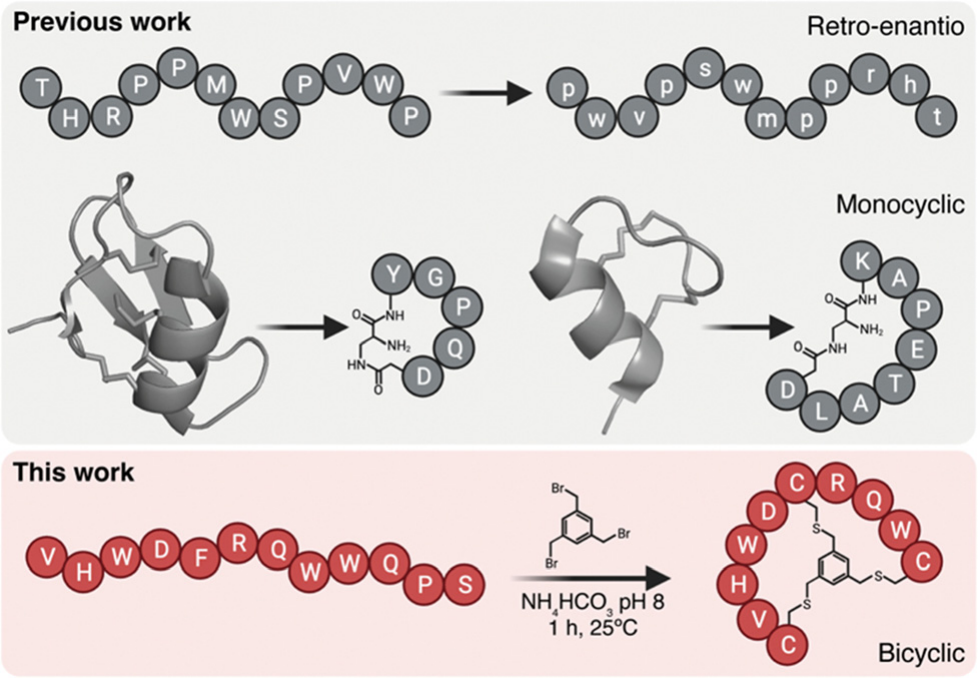
BrainBike bicyclic peptidomimetics are a new class of brain shuttles. The top panel shows the retro-enantio strategy applied to the THR peptide^6^ and the development of monocyclic peptides MiniAp-4 and MiniCTX3 from the cysteine-rich peptides apamin and chlorotoxin, respectively.^10,11^ The bottom panel depicts the strategy developed in the present work, the transformation of linear Y1 peptide into bicyclic BrainBike-4.

Here we generate a new family of bicyclic brain shuttles, BrainBikes or BBs, with the capacity to enhance the transport of therapeutic proteins across brain endothelium (Fig. 1 and 2A). We design and synthesize several BrainBikes from a linear peptide binding the Transferrin Receptor 1 (TfR1), a receptor known to mediate RMT on the BBB. First, we prove that all analogs are more protease resistant than the parent peptide. Then, we demonstrate that some of the analogs display enhanced binding to TfR1 and to cells overexpressing this receptor. Next, we conjugate the peptide site-specifically to a green fluorescent protein and a single domain antibody variable fragment (scFv). Finally, we show that the bicyclic peptide provides a 4-to-5-fold transport enhancement across human brain endothelium.^2^

**Fig. 2:**
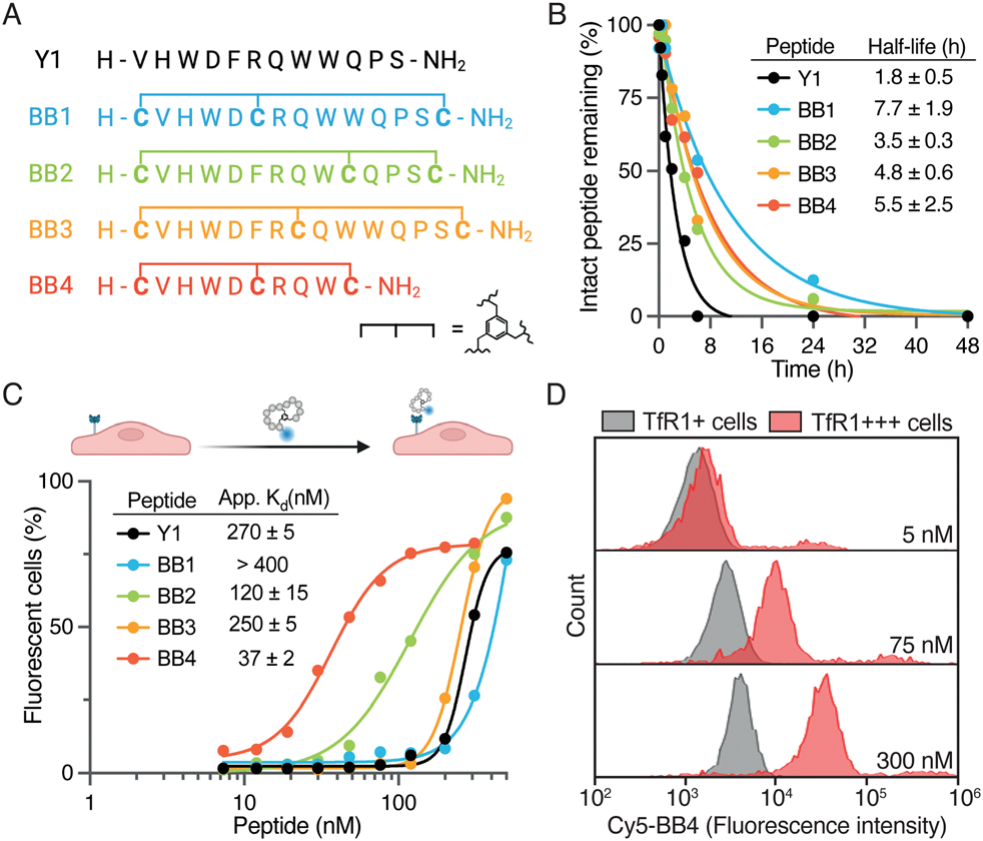
(A) Sequences of the bicyclic analogs designed here. (B) Stability in human serum of Y1 and BrainBikes. The percentage of intact peptide in serum as assessed by HPLC-UV, and confirmed with mass spectrometry, depicted as a function of time. (C) Association of Y1 and bicyclic analogs to HeLa cells (TfR1+), as measured by flow cytometry. (D) Association of BrainBike-4 to HeLa cells (TfR1+) and HeLa cells transfected with TfR1 (TfR1+++). For the complete set of data see Fig. S4C (ESI†).

We selected TfR1 as the target for BrainBikes because this receptor is highly expressed in the brain endothelium and has extensively been exploited for the delivery of large therapeutics using antibody brain shuttles.^2^ However, protease-resistant peptide shuttles reported until now that utilize this receptor have very limited capacity to transport protein cargoes across endothelial cells.^11,17^ As a starting sequence for the shuttles, we selected the 12-mer peptide Y1 recently reported by Tan and coworkers.^18^ Y1 was identified *via* phage display panning against the extracellular domain of TfR1 and showed high selectivity for cancer cells overexpressing TfR1. In addition, Y1 was shown not to compete with endogenous transferrin for binding to its receptor, which should maximize transport efficiency. We devised four bicyclic analogs of Y1, *i.e.* BrainBike 1 to 4 (BB 1 to 4) (Fig. 2A). Although bicyclic peptides may be selected *via* phage display, they can also be generated from linear sequences by rational design.^19^ All BrainBikes contain one cysteine at each termini and one additional cysteine toward the center of the sequence that will react with a 1,3,5-tris(bromomethyl)benzene (TBMB) linker. Once the central cysteine is conjugated to TBMB, this position will become highly hydrophobic. Thus, in the first two analogs we replaced hydrophobic residues, either Phe5 or Trp9, by a cysteine. In the third analog, we inserted the cysteine equidistant from both termini, leaving two cycles of six residues. In the fourth and last analog, we replaced Phe5 and cropped the C-terminal residues to generate a more strained and compact bicycle. The parent peptide and the bicyclic analogs were synthesized *via* Fmoc/^*t*^Bu solid-phase peptide synthesis (Table S1 and Fig. S1, ESI†). Peptides were characterized by HPLC-UV and mass spectrometry, and the secondary structure was assessed *via* circular dichroism. We observed that in BrainBike-1 and BrainBike-3 the slight tendency to form an alpha helical structure was preserved with respect to the parent peptide, while in BrainBike-2 and BrainBike-4 it was practically inexistent (Fig. S2, ESI†).

Peptides often present two drawbacks for pharmacological applications: low residence time in blood and low resistance to proteolysis. While fast renal clearance is not a main issue when conjugated to large cargoes such as therapeutic proteins, low resistance to proteases often represents a major weakness.^5^ We and others have shown that resistance to proteases is key for efficient brain transport, since it enables improved interaction with receptors on the brain endothelium and transit across the BBB.^4,6^ Therefore, we studied the stability of the peptides in human serum for 48 h. Although Y1 displayed relatively high stability for a linear peptide, with a half-life of 1.8 h (Fig. 2B), all BrainBikes presented enhanced stability in serum, as expected. Bicyclization leads to a 2- to 4-fold increase in half-life, which is reasonable taking into account the length of the loops. Although degradation of the linear peptide starts at the N-terminus (Fig. S3, ESI†), endoproteases also must play a key role in the proteolysis since shielding the termini *via* cyclization is not sufficient to provide full protection.

After confirming higher metabolic resistance of the bicyclic analogs, we aimed to study whether rigidification might also result into higher receptor affinity. To this end, we assessed the capacity of peptides to bind to HeLa cells. These cells express relatively high levels of TfR1 (Fig. S4A, ESI†) and were also used in the publication reporting the parent peptide.^18^ To study peptide binding by flow cytometry we conjugated sulfo-Cyanine5 *N*-hydroxysuccinimide activated ester (sCy5-NHS) to the N-terminal amine of the peptides. Most BrainBikes were able to retain similar binding to the parent peptide (Fig. 2C). This indicates that the hydrophobic residues replaced are not involved in binding and that the relatively long loops enable sufficient flexibility to adopt the binding conformation in all analogs. Of note, two peptides displayed substantially higher cell association than the parent peptide: BrainBike-2 and BrainBike-4, by 2- and 7-fold, respectively (Fig. 2C). In both analogs, Trp9 is replaced by a cysteine conjugated to the trifunctional linker, which seemingly induces a change in conformation that decreases its tendency to adopt an alpha-helical structure. Since the analogs with highest affinity lack the alpha-helical character (Fig. S2, ESI†), it would appear this feature is not relevant for binding. BrainBike-4 is the analog displaying highest affinity, with a dissociation constant on cells of roughly 40 nM. This peptide is also the one with the most constrained loops, presumably preorganizing more efficiently the peptide binding conformation. The four C-terminal residues that have been removed in this analog appear not to be involved in the interaction with the target receptor.

Next, we verified that the association of BrainBike-4 to HeLa cells was due to the TfR1 by overexpressing this receptor in the same cell line (Fig. 2D). We transfected HeLa cells with a plasmid encoding for TfR1 fused to a green fluorescent protein (GFP-TfR1) so that we could analyze binding to the cells expressing higher levels of the receptor. We confirmed TfR1 was overexpressed on transfected cells using a commercial anti-TfR1 antibody (Fig. S4B, ESI†). Remarkably, binding of BrainBike-4 to cells overexpressing TfR1 was roughly 40-fold higher than to the same cells without overexpressing the receptor (Fig. S4C, ESI†). A final proof of the shuttle binding to TfR1 was obtained by biolayer interferometry with the immobilized extracellular domain of the receptor (Fig. S5A, ESI†). Tighter binding of BrainBike-4 with respect to the parent peptide Y1 was confirmed both on cells (Fig. S4C, ESI†) and on the extracellular domain of TfR1 (Fig. S5, ESI†).

With the lead candidate BrainBike-4 in hand, we sought to challenge its capacity to transport proteins across brain endothelium. We selected two cargoes: the green fluorescent protein (GFP) as a common model cargo and an antibody derivative as a therapeutically relevant protein. Antibodies display high efficacy in peripheral tissues in many types of cancer. However, these macromolecules have very limited permeability across the BBB due to their large size. There is increasing interest in antibody derivatives due to their deeper tissue penetration and higher engineering versatility.^20^ Therefore, we selected a single chain antibody variable fragment (scFv) as the cargo to be transported.^21,22^

Site-specific anchoring of a brain shuttle to a full-length antibody has recently been shown to provide increased brain accumulation compared to random conjugation.^23^ Moreover, we have previously shown that the conjugation site of the shuttle has a significant impact on the shuttle efficiency.^24^ Therefore, we aimed to generate homogeneous conjugates with our best brain shuttle, BrainBike-4. To link this peptide to GFP we utilized thiol-maleimide chemistry as it has been reported for other reference shuttles.^11,17^ A cysteine was encoded near the C-terminus of the protein and it was quantitatively reacted with a maleimide-bearing BrainBike-4 (Fig. 3A and Fig. S6C and S7, ESI†). For the controlled conjugation of the brain shuttle to the scFv, we utilized a chemoenzymatic approach that yielded a stable linker for future studies *in vivo* (Fig. 3D). We selected the C-terminus of the scFv as the anchoring site since it is distant from the binding region and its modification should not affect antigen engagement. We first incorporated a linker bearing a bicyclononyne (BCN) bioorthogonal handle to the C-terminus of the scFv using the transpeptidase sortase A.^25^ Since the sortase A substrate was encoded prior to the purification tag, this tag was removed and replaced by the Gly-poly(ethyleneglycol)-BCN linker, resulting into a slight decrease in molecular weight (Fig. 3E and Fig. S8, ESI†). The reaction was quantitative and yielded the expected scFv(BCN) as assessed by mass spectrometry (Fig. 3E and Fig. S6, ESI†). We then conjugated the BCN handle on the scFv with an azide group previously incorporated at the N-terminus of BrainBike-4 on solid phase synthesis. The BCN on the scFv reacted with the azide on the peptide *via* a strain-promoted azide–alkyne cycloaddition. Conjugation of the peptide was quantitative within 1 h, as verified by mass spectrometry and SDS-PAGE (Fig. 3E and Fig. S6 and S8, ESI†). In this way, we obtained an scFv, with a defined ratio of one peptide per protein, to be used in the transport assays. We also prepared the scFv conjugate with Angiopep-2 as a reference brain shuttle.^26^

**Fig. 3:**
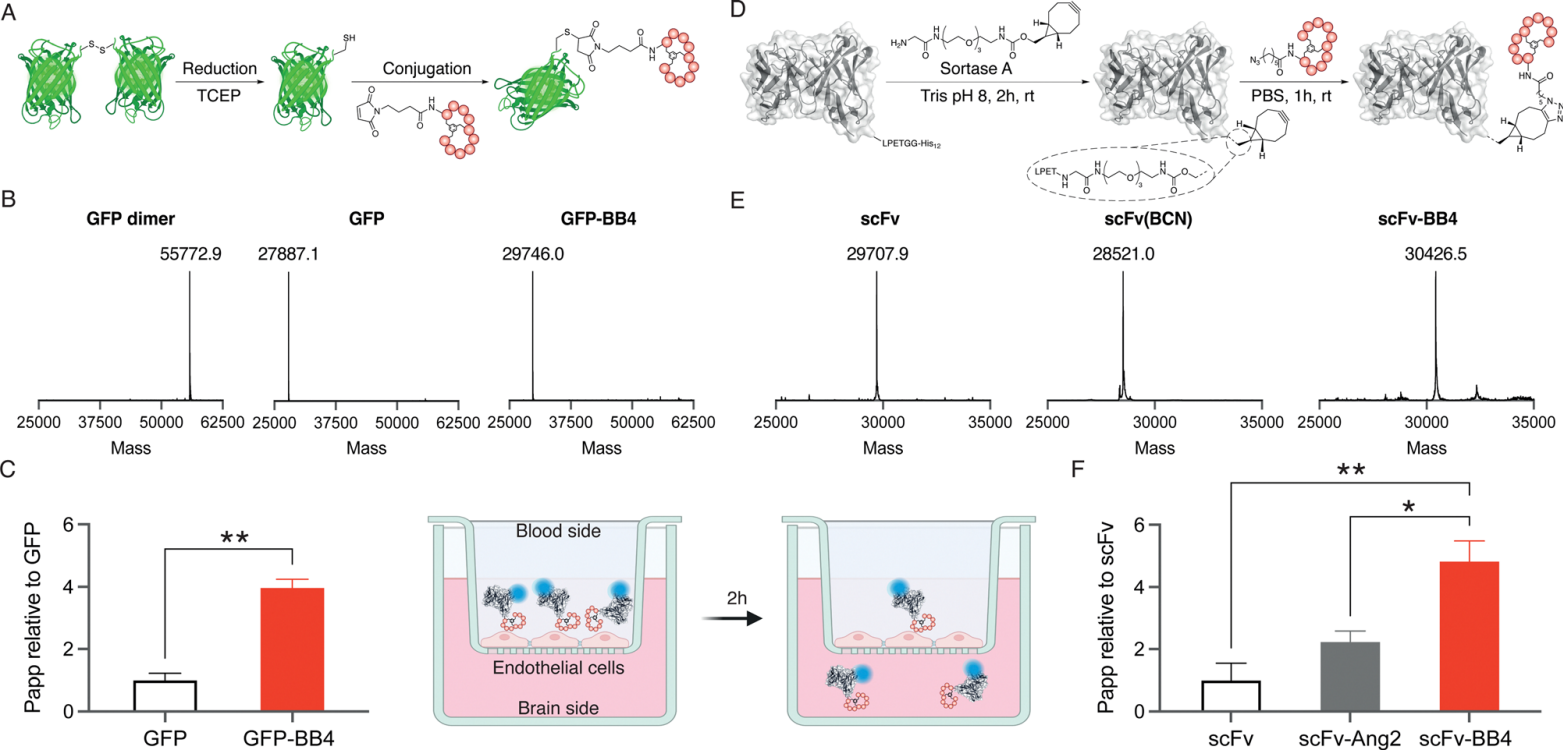
Representation of the conjugation of BrainBike-4 to (A) GFP and (D) scFv. (B) and (E) Deconvoluted mass spectra of the six constructs represented above. Transport across a tight human brain endothelial layer mimicking the human blood–brain barrier of (C) GFP and its BB-4 conjugate, (F) scFv and its BB-4 and Ang2 conjugates. Error bars indicate the standard error of the mean (*n* = 3; *p < 0.01, **p < 0.001).

Finally, the permeability of the protein-shuttle conjugates was studied in a human cell-based model of the BBB. We labelled the scFv conjugate with sCy5-NHS to enable usage of low concentrations of proteins (nM) to avoid saturation of the receptors on endothelial cells. An average of two sCy5 molecules per antibody were conjugated (Fig. S8, ESI†). The BBB model used was based on a tight monolayer of human brain endothelial cells seeded on Transwell® membranes. This model is robust, shows a good correlation with *in vivo* permeability, and is well validated for expression of BBB markers, including the transferrin receptor.^27,28^ The integrity of the model was verified with an internal standard of lucifer yellow (Table S2, ESI†). Remarkably, BrainBike-4 enables a 4-fold increase in the transport of GFP and a 5-fold increase in the transport of the scFv (Fig. 3C and F and Table S2, ESI†). The transport enhancement enabled by BrainBike-4 is substantially higher than the 1.4- to 2-fold increase we have previously reported with other protease-resistant peptide RMT shuttles, also conjugated to GFP and assayed in the same model.^10,11^ Furthermore, the transport of scFv-BB4 is significantly higher (*p* <0.01) than the transport the scFv conjugated to Angiopep-2, the current gold-standard peptide brain shuttle.

Overall, we have created a new class of bicyclic brain shuttle peptidomimetics, BrainBikes. We have shown that linear peptide shuttles binding TfR1 can be turned into BrainBikes with enhanced protease resistance and affinity for TfR1. The lead shuttle BrainBike-4 site-specifically conjugated to GFP or an scFv increased the transport of these proteins in a human cell-based model of the BBB several times more than previously reported shuttles. The high transport capacity of BrainBike-4 makes it a promising shuttle for the efficient delivery of biotherapeutics across the BBB. Furthermore, this study expands the current toolset to generate new brain shuttles for the wide variety of therapeutic cargoes that will benefit from an enhanced transport into the brain.

B. O.-S. conceptualized the project. M. C. L., C. D.-P., and B. O.-S. designed all experiments. R. L. contributed the scFv production and conjugation. M. C. L. and C. D.-P. performed all other assays. F. G. contributed the BBB model. B. O.-S., C. D.-P., and M. C. L. wrote the paper with contributions from all authors.

## Conflicts of interest

There are no conflicts to declare.

## Supplementary Material

CB-005-D3CB00194F-s001
