# Supplementary material for: BrainBike peptidomimetic enables efficient transport of proteins across brain endothelium
Source: RSC Chem Biol. 2023 Dec 11;5(1):7–11. doi: 10.1039/d3cb00194f (PMC10763564; doi:10.1039/d3cb00194f)
Supplement: CB-005-D3CB00194F-s001 [file CB-005-D3CB00194F-s001.pdf]

Electronic Supplementary Material (ESI) for RSC Chemical Biology.
This journal is © The Royal Society of Chemistry 2023

Materials and methods

Peptide synthesis and characterization

Solvents and reagents

Solvents and reagents were obtained from commercial suppliers such as Iris Biotech (Marktredwitz, Germany), Sigma-Aldrich (Milwaukee, WI), Carlo Erba Reagents (Emmendingen, Germany), Lumiprobe (Hannover, Germany), Broadpharm (California, USA), and were of the highest purity commercially available. Protected amino acids were supplied by Iris Biotech and SDS (Barcelona, Spain). RinkAmide ChemMatrix® resin was purchased from PCAS BioMatrix Inc. (Quebec, Canada). Syringes, eppendorfs and falcons were acquired from Scharlau (Barcelona, Spain) and Deltalab. Cell culture-treated plates and flasks were purchased from Corning Costar. Culture medium was acquired from Lonza. Desalting columns (MiniTrap and MidiTrap G-25) were obtained from GE-Healtcare.

Solid-phase peptide synthesis

Peptides were assembled using manual Fmoc/tBu solid-phase peptide synthesis (SPPS). The peptides were synthesised in a 200 µmols scale using Rink amide (aminomethyl)polystyrene resin (substitution of 0.70 mmol/g). Synthesis was performed on polypropylene syringes with a polypropylene porous filter. The stirring was done manually, intermittently, with a Teflon stirring bar and reagents and solvents were eliminated under vacuum filtration. Amino acids were coupled to the growing chain using standard protocols. Briefly, the resin was washed several times and the temporary protecting group Fmoc was removed using a solution of 20% piperidine in DMF. The default method for incorporating all the amino acids into the chain consisted of a neutral activation using 4 eq. of diisopropylcarbodiimide (DIC) and 4 eq. of OximaPure® employing 4 eq. of the respective amino acid. The reaction was performed on DCM/DMF 1:1 for 1 h. For the incorporation of an azide or maleimide moiety at the N-terminal position, 3 eq. of 6-azido hexanoic acid were added to the resin with 3 eq. of DIC and 3 eq. of OximaPure® in DMF and left to react for 1h. After the washings of the resin from the reagents and by-products, the coupling control was carried out by the ninhydrin test, to detect primary amines, or chloranil test, for the detection of secondary amines.

Cleavage from the resin/side-chain deprotection

After the deprotection of the *N*-terminus of the last amino acid, the resin was washed several times with DMF and DCM and dried by suction for 15 minutes. The dried resin was transferred to a 50 mL falcon and the peptides were cleaved from the resin using trifluoroacetic acid (TFA), H_2O and triisopropylsilane (TIS) in the following proportions 92.5:2.5:2.5 % (v/v). When protected cysteines were present in the sequence, the cocktail contained TFA, ethanedithiol (EDT), H_2O and TIS in the following concentrations 94:2.5:2.5:1 % (v/v). The resin was

incubated for 3 h in the cocktail cleavage. Then, the solvent was evaporated applying an argon current. Diethyl ether was added at 0°C to precipitate the peptide and eliminate non-peptidic impurities, and the mixture was centrifuged (4°C, 5000g, 5 min). The supernatant liquid was decanted and the process was repeated three times. After that, the cleaved peptides were dissolved in H_2O/MeCN (1:1, 0.1% TFA) and filtered off the resin. Finally, peptides were lyophilised.

Linker incorporation and cyclization

The cyclization in solution was based on Heinis *et al* protocol[1] the crude peptide (0.5 mM) in 70% (v/v) 10 mM NH_4HCO_3 pH 8 and 30% (v/v) MeCN was reacted with 1,3,5-tris(bromomethyl)benzene (TBMB) (0.6 mM) for 1 h at 25°C. The correct cyclization was assessed using MALDI-TOF.

Conjugation of peptides to sulfo-cyanine5 N-hydroxysuccinimide (sCy5-NHS) ester

After peptide cleavage and cyclization, the conjugation to sCy5-NHS fluorophore was performed in anhydrous medium to avoid the hydrolysis the NHS moiety. 1 eq. of the crude peptide was treated with 1.5 eq. of fluorophore and 4 eq. of DIPEA in anhydrous DMSO. The mixture was allowed to react for 30 min and the completion of the reaction was assessed by HPLC and MALDI-TOF. Ethanolamine was used to quench the unreacted esters.

Purification of peptides

Peptides were dissolved in H_2O/MeCN (the percentage depending on the peptide) and filtered through 0.45 µm filters. Then, they were purified on an Agilent 1260 Infinity II system with ChromScope software, a 1260 VWD, a 1260 Preparative Binary Pump, a 1260 Column Organizer and a 1290 Preparative Fraction Collector. An Aeris Peptide XB-C18 100 LC Column (250 x 10 mm, 5 µm, Phenomenex) was used, with MeCN (0.1% TFA) and H_2O (0.1% TFA) as solvents and a flow rate of 12 mL/min. The fractions containing the peptide were analysed by MALDI-TOF, pooled together and lyophilised.

Peptide characterization

Analytical UHPLC

UHPLC-UV chromatograms were obtained on an Acquity 1260 Infinity II system with ChromoScope software (1260 DAD WR, 1260 Vial Sampler and 1260 Flexible Pump) using an Acquity BEH C18 (50 x 2 mm x 1.7 µm) column. The flow rate was 1 mL/min using a standard gradient of 5-95% MeCN (0.1% TFA) in water (0.1% TFA) in a 12 min linear gradient.

MALDI-TOF

A MALDI-TOF Bruker Biotyper MBT smart was used to determine the molecular weight of the peptides. Samples were prepared on a MALDI-TOF plate (Anchor Chip 96) by mixing 0.5 µL of peptide solution and 0.5 µL of α- ciano-4-hydroxycinamic acid (ACH). The ACH matrix was prepared at 10 mg/mL in MeCN/H_2O 1:1 (v/v; 0.1% TFA).

Circular dichroism

Spectra were recorded using a Jasco 815 UV-Vis spectropolarimeter. Peptide samples were dissolved in 15 mM Na_2HPO, 5 mM NaH_2PO_4, pH 7.2, and the spectra were recorded at a 100 µM concentration. The following parameters were used: start (260 nm), end (190 nm), scanning mode (continuous), scanning speed (200 nm/min), response (2s), band width (2.0 nm). A blank spectrum of the buffer was subtracted from all recordings.

Peptide stability in human serum

Peptides were dissolved in human serum at a final concentration of 100 µM and then incubated at 37°C. Aliquots of 50 µL were taken at different times (0 min, 15 min, 30 min, 1 h, 2 h, 4 h, 6 h, 24 h and 48 h) and flash frozen with acetone/solid CO_2. Samples were treated with 200 µL of MeOH to precipitate serum proteins. Samples for UHPLC analysis were centrifuged and filtered through a 0.22 µm filter before injection. Aliquots were analysed with UHPLC-UV (flow = 0.61 mL/min; gradient 5-95% MeCN (0.036% TFA) in water (0.045% TFA) in 2 minutes). The amount of peptide remaining was calculated by comparing the area under the curve of the several samples.

Protein production, conjugation, and characterization

Protein production and conjugation

Expression of the Cys-GFP

E. coli BL21 cells were transfected by heat shock with pET29 plasmid carrying the gene for GFP expressing an extra cysteine at the N-terminus. One single colony was used to inoculate 500 mL of LB media, containing kanamycin (50 µg/mL) and the culture was grown overnight at 150 rpm at 37°C. Cells were harvested by centrifugation at 6000 g for 15 min at 4 °C. Pellets were resuspended in lysis buffer (50 mM TRIS-HCl pH=8.0, 150 mM NaCl, 1% Triton X-100 and 1 mM PMSF) and intracellular content was released by sonication at 40% amplitude, with 25 s ON and 30 s OFF pulses, for a total of 10 min on ice. Lysis suspension was clarified centrifuging at 25000 g for 25 min at 4 °C. The protein was purified by Immobilised Metal Chelate Affinity Chromatography (1 mL IMAC HisTrap™ HP column from Cytiva) on a fast protein liquid chromatography system (FPLC, BIO-RAD NGC). Fractions containing the protein were pooled.

Expression of the anti-CD133 scFv antibody

The production of the anti-CD133 scFv antibody was performed following an adapted version of the protocol described in Swaminathan *et al*. 2013.[2] The plasmid encoding the scFv, supplied by Prof. Jayanth Panyam (University of Minesotta), was modified to incorporate a Sortase A recognition sequence at the C-terminal and a His_{12} tag.

Sortase A ligation of the BCN linker to the scFv

Once the antibody was produced, the reaction with Sortase A (SrtA, BPS Bioscience Catalog #71046, or in-house produced from Addgene Catalog #75144) to remove the His-tag and incorporate the BCN linker (Gly-PEG_3-endo-BCN, BroadPharm, Catalog #BP-24232) was performed. 5 eq. of SrtA and 10 eq. of BCN linker were added to 1 eq. of scFv (35 µM) in 50 mM Tris-HCl, 150 mM NaCl and 0.5 mM $CaCl_2$. The reaction was allowed to proceed for 2 h at 22°C. Then, SrtA and the unreacted antibody were purified from the conjugate in an IMAC nickel-loaded column, where the BCN-modified antibody eluted in the flow-through. The purity of the product was assessed by SDS-PAGE and LC-QTOF.

Conjugation of the brain shuttles to the proteins and antibodies and labelling

Conjugation of the peptides to Cys-GFP

The conjugation of GFP incorporating an extra cysteine with the maleimido-labelled peptide was accomplished by mixing the two components in a 1:25 ratio in PBS buffer. They were left to react for 2h, and the progress of the reaction was assessed by LC-MS and SDS-PAGE.

Conjugation of the peptides to the anti-CD133 scFv antibody

The conjugation of the anti-CD133 scFv antibody incorporating the BCN moiety (previously labelled with the fluorophore) with the azide-labelled peptide was accomplished by mixing the two components in a 1:25 ratio in PBS buffer. They were left to react for 1h, and the progress of the reaction was assessed by SDS-PAGE.

Labelling of anti-CD133 scFv antibody with sulfo-Cyanine5 NHS ester

One eq. of anti-CD133 antibody (50 µM stock concentration) in PBS was treated with 10 eq. of sCy5 NHS ester (10 mM stock concentration in DMSO). The mixture was allowed to react for 60 min and the excess fluorophore was removed by dialysis (Slide-A-Lyzer™ MINI Dialysis Devices, 2K MWCO, ThermoFisher) against PBS following the manufacturer's protocol (dialysing for 15 min and changing the buffer four times). The correct conjugation was assessed by SDS-PAGE and LC-QTOF.

Protein characterization

SDS-PAGE

SDS-PAGE electrophoresis was carried out using BioRad system (Miniprotean cell) in a 15% Tris gel using the following running buffer: 1.92 M Gly, 0.25 M Tris, 1% SDS, pH 8.3. The gel run at 180 V until the marker reached the bottom of the gel. Protein molecular weights were approximated by comparison to a protein marker (Precision Plus Protein Dual Color Standards from BioRad). Proteins were visualized by Coomassie staining (staining solution: 10% AcOH, 0.25 g brilliant blue; unstaining solution: 40% MeOH, 10% AcOH in water).

LC-QTOF

Samples were analysed using the Q-TOF X500B connected to an Exion LCAD chromatograph. The peptide analysis was performed using a column Acquity BEH C18 (50 x 2 mm x 1.7 µm). applying a 5-95% gradient MeCN (0.1% FA) in water (0.1% FA) in 5 min. Q-TOF MS was operated in the positive ion mode for the analysis. The mass spectra were recorded across the range of 100–3000 Da with a fixed collision energy of 10 V. Data acquisition and evaluation was performed using SCIOX OS software.

Cell experiments

Cell culture

HeLa cells were cultured in DMEM media (1000 mg/L glucose) supplemented with 10% fetal bovine serum (FBS), 1% of 2 mM L-glutamine and 1% antibiotics (100 units/mL of penicillin and 100 µg/mL of streptomycin). Cells were expanded at 80% confluence and grown at 37°C with 5% CO_2. All work performed with human cells follow the ethical principles and EU Directive 2004/23/EC.

Cell transfection of HeLa cells to express higher levels of TfR1

In a 96-well plate, 10,000 HeLa cells per well were seeded and left to grow for 24 h. A mixture of Lipofectamine 3000 (0.1 µL/well, Lipofectamine™ 3000 Transfection Reagent, ThermoFisher Scientific), P3000 Reagent (0.2 µL/well) and the DNA pTfR-mNeonGreen (Plasmid #129608, Addgene) (100 ng/well) in Opti-MEM medium was prepared. The mixture was incubated for 15 min, added to the cells, and incubated for 48 h.

Cell binding assays

48-hour post-transfection, 50 µl of the sulfo-Cy5-labelled peptides were added to each well at the appropriate concentration (from 5 to 600 nM) and incubated at 4°C for 1 hour. Cells were washed three times with 100 µL of PBS, trypsinized with 25 µl of trypsin for 5 min at 37°C and, then 75 µL of supplemented medium with formalin (33% v/v) was added. Cells were detached by repeated pipetting and analysed using an Agilent NovoCyteflow cytometer (Agilent). sCy5 was analysed at 640 nm while mNeon-Green at 488 nm. Data was analysed by plotting the geometric mean vs the concentration of the peptide.

Permeability assays in the in vitro BBB cellular model

These experiments were performed using the model developed by Cecchelli and collaborators.[3] Briefly, endothelial cells derived from pluripotent stem cells (brain like endothelial cells) and bovine pericytes were defrosted in gelatin-coated Petri dishes (Corning). Pericytes were cultured in DMEM pH 6.8 while endothelial cells were cultured in supplemented endothelial cell growth medium (sECM) (Sciencells). After 48 h, endothelial cells were seeded in 0.4 µm polycarbonate 12-well Transwell inserts (8,000 cell/well) and pericytes were plated in 12-well plates (50,000 cells/well) previously coated with Matrigel and gelatin, respectively. The medium was changed every 2-3 days and the assays were performed 7-8 days after seeding by placing the transwells into new wells without pericytes. 100 nM of protein or antibody conjugates was used in each well. Lucifer Yellow (50 µM) was added as a control of barrier integrity (Papp < $17{\cdot}10^{-6}$ cm/s). To perform the assay, 500 µL of the compound in Ringer HEPES was added to the donor compartment and 1500 µL of Ringer HEPES was introduced into the acceptor compartment. The plates were incubated for 2 h at 37°C, and the solutions from both compartments were recovered and analysed. The samples were evaluated in triplicates. The amount of protein conjugates was quantified by fluorescence with Fluorescence Spectrophotometer F2500 HITACHI. The selected slit for excitation and emission was 5 nm and 10 nm, respectively. The voltage for the detector was 700 V. Apparent permeability was calculated with the following formula:

$$P_{app} = \frac{Q_A(t) \cdot V_D}{A \cdot Q_D(t_0)}$$

where P_{app} is obtained in cm/s, $Q_A(t)$ is the amount of compound at the time t in the acceptor well, V_D is the volume in the donor well, t is time in seconds, A is the area of the membrane in cm and $Q_D(t_0)$ is the amount of compound in the donor compartment at the beginning of the experiment.

Biophysical characterization

Biolayer Interferometry

Binding assays were performed on an Octet R2 (Sartorious) using streptavidin-coated biosensors (SA biosensors, 18-5019). Biosensors were equilibrated for at least 10 min in binding buffer (10 mM HEPES pH 7.4, 150 mM NaCl, 0.05 % Tween-20, 1 mg/mL BSA). Experiments were designed and executed from the Octet BLI Discovery 13.0 software. The biotinylated human Transferrin Receptor (TFR-H82E5, Acro Biosystems) was immobilized onto the biosensor by dipping the sensors into a solution of 1 µg/mL hTfR for 500 s, followed by dipping the sensor into a fresh solution of binding buffer to stablish a baseline for 200 s. Titrations were executed at 25ºC while rotating at 1000 rpm. In the association phase, the sensors were dipped into a solution containing the peptides at several concentrations in binding buffer for 150 s. After reaching equilibrium, the biosensors were dipped into fresh binding solution to monitor the dissociation kinetics for 200 s. Kinetic data were collected and processed using a 1:1 binding model to obtain the affinity constants using the Octet Analysis Studio 13.0.

Statistical analysis

Unpaired two-tailed student t tests were applied to evaluate the significant difference or p values between data sets using Prism 6.0c software.

References

1. C. Heinis, T. Rutherford, S. Freund and G. Winter, *Nat Chem Biol*, 2009, **5**, 502–507.
2. S. K. Swaminathan, L. Niu, N. Waldron, S. Kalscheuer, D. M. Zellmer, M. R. Olin, J. R. Ohlfest, D. A. Vallera and J. Panyam, *Drug Deliv Transl Res*, 2013, **3(2)**, 143–151.
3. R. Cecchelli, S. Aday, E. Sevin, C. Almeida, M. Culot, L. Dehouck, C. Coisne, B. Engelhardt, M. P. Dehouck and L. Ferreira, *PLoS One*, 2014, **9**, e99733.

Supplementary Tables and Figures

Table S1 Sequence, molecular formula, molecular weight (MW), UPLC characterisation (t_R, retention time) and purity after synthesis and purification of peptides

Peptide name	Calc MW	$[M+H]^+$ MALDI-TOF MS	t_R, UHPLC, min	Purity, %	Sequence
GY1	1727.8	1728.9	5.91	> 98	H-GVHWDFRQWWQPS-NH_2
BrainBike-1	1947.9	1949.0	5.39	95	H-CVHWDCRQWWQPSC-NH_2 (TBMB)
BrainBike-2	1908.9	1909.7	5.63	> 98	H-CVHWDFRQWCQPSC-NH_2 (TBMB)
BrainBike-3	2095.0	2096.0	5.89	> 98	H-CVHWDFRCQWWQPSC-NH_2 (TBMB)
BrainBike-4	1449.7	1449.7	5.27	> 98	H-CVHWDCRQWC-NH_2 (TBMB)
sCy5-GY1	2350.7	2353.3	6.30	> 98	sCy5-GVHWDFRQWWQPS-NH_2
sCy5-BrainBike-1	2571.0	2572.7	6.00	> 98	sCy5-CVHWDCRQWWQPSC-NH_2 (TBMB)
sCy5-BrainBike-2	2533.0	2534.6	6.20	> 98	sCy5-CVHWDFRQWCQPSC-NH_2 (TBMB)
sCy5-BrainBike-3	2718.2	2719.0	6.30	90	sCy5-CVHWDFRCQWWQPSC-NH_2 (TBMB)
sCy5-BrainBike-4	2274.6	2275.4	5.90	> 98	sCy5-GSGCVHWDCRQWC-NH_2 (TBMB)
N_3-BrainBike-4	1790.0	1791.1	6.28	> 98	N_3-GSGCVHWDCRQWC-NH_2 (TBMB)
Mal-BrainBike-4	1857.7	1857.7	6.40	>98	Mal-GSGCVHWDCRQWC-NH_2 (TBMB)
N_3-Ang2	2440.7	2442.7	6.23	> 98	N_3-TFFYGGSRGKRNNFKTEEY-NH_2

Mal = 3-(Maleimido-1-yl)propanoic acid
N_3 = 6-azido-hexanoic acid
sCy5 = sulfo-cyanine-5

Table S2 BBB-model permeabilities. All values are Mean± SD

Sample	Permeability (cm/s)	Relative permeability	LY permeability (cm/s)
GFP	$6.4\cdot10^{-8} \pm 2.6\cdot10^{-8}$	1.0 ± 0.4	$9.9\cdot10^{-6}$*
GFP-BrainBike-4	$2.7\cdot10^{-7} \pm 3.3\cdot10^{-8}$	4.0 ± 0.5	$9.9\cdot10^{-6}$*
scFv	$3.4\cdot10^{-7} \pm 3.2\cdot10^{-7}$	1.0 ± 0.9	$8.9\cdot10^{-6}$
scFv-Ang2	$7.6\cdot10^{-7} \pm 2.1\cdot10^{-7}$	2.2 ± 0.6	$8.0\cdot10^{-6}$
scFv-BrainBike-4	$1.7\cdot10^{-6} \pm 3.1\cdot10^{-7}$	4.8 ± 1.1	$7.4\cdot10^{-6}$

*Measured in three independent wells without GFP protein incubation

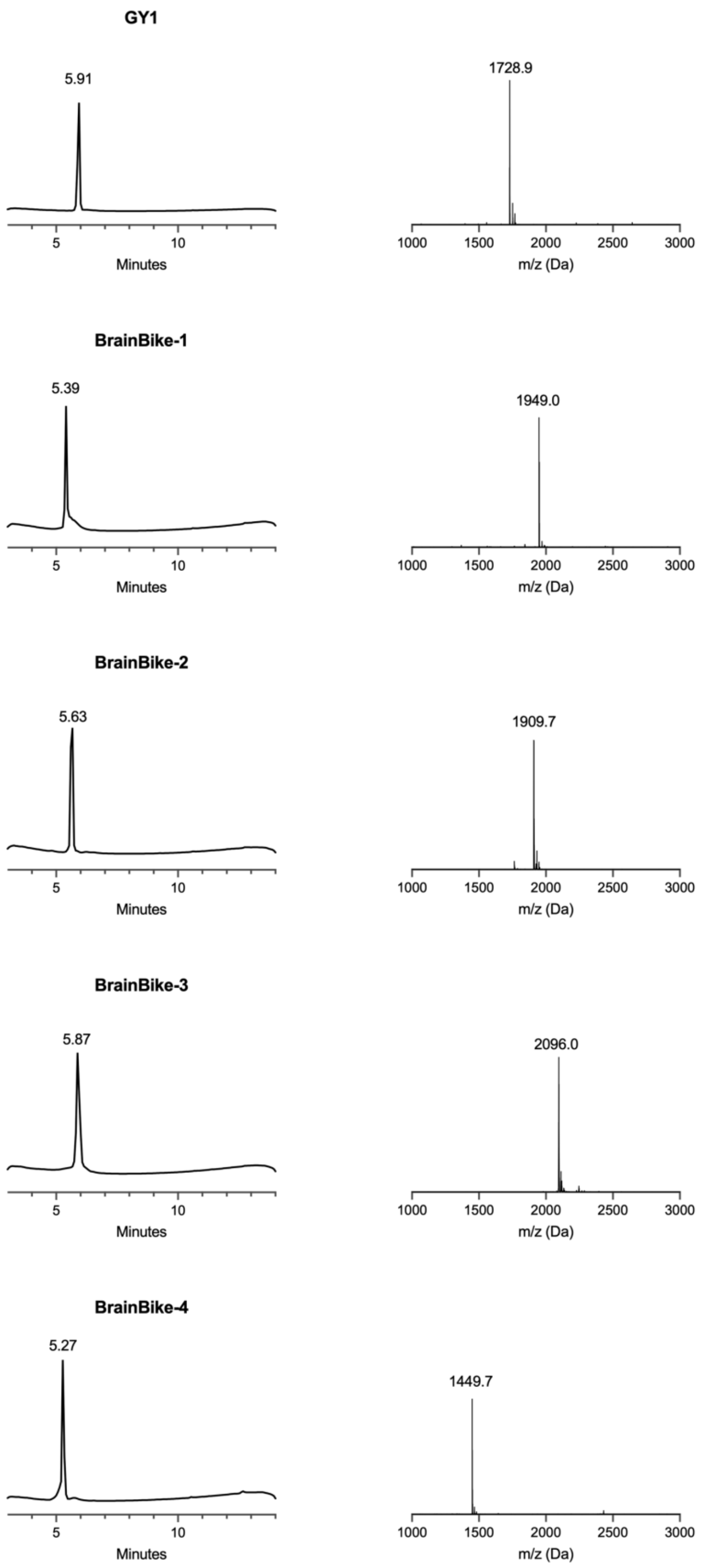

Figure S1 Peptide characterization. Chromatograms (left) and mass spectra (right). Chromatograms were obtained using a gradient of 5-95% in 12 min of acetonitrile 0.1% TFA in water 0.1% TFA.

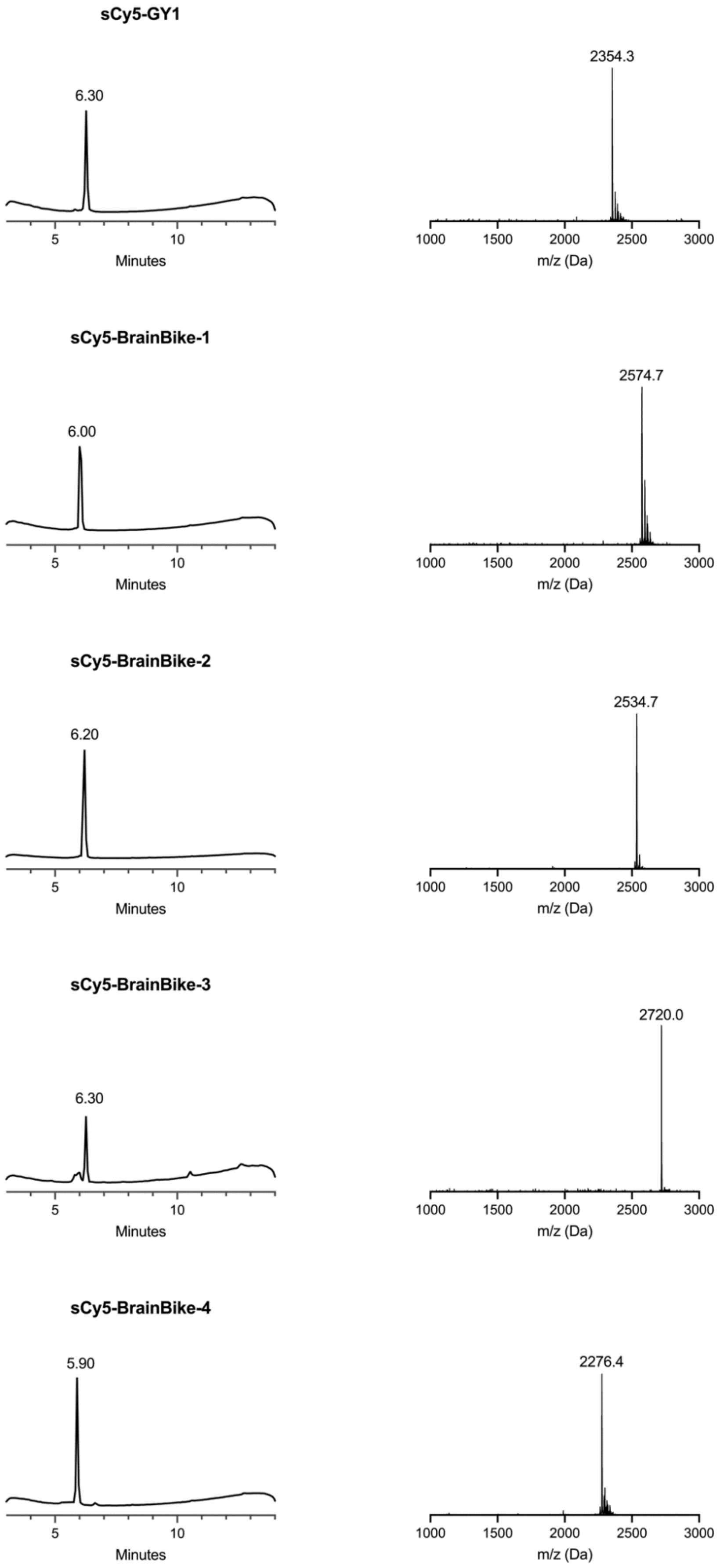

Figure S1 Continued

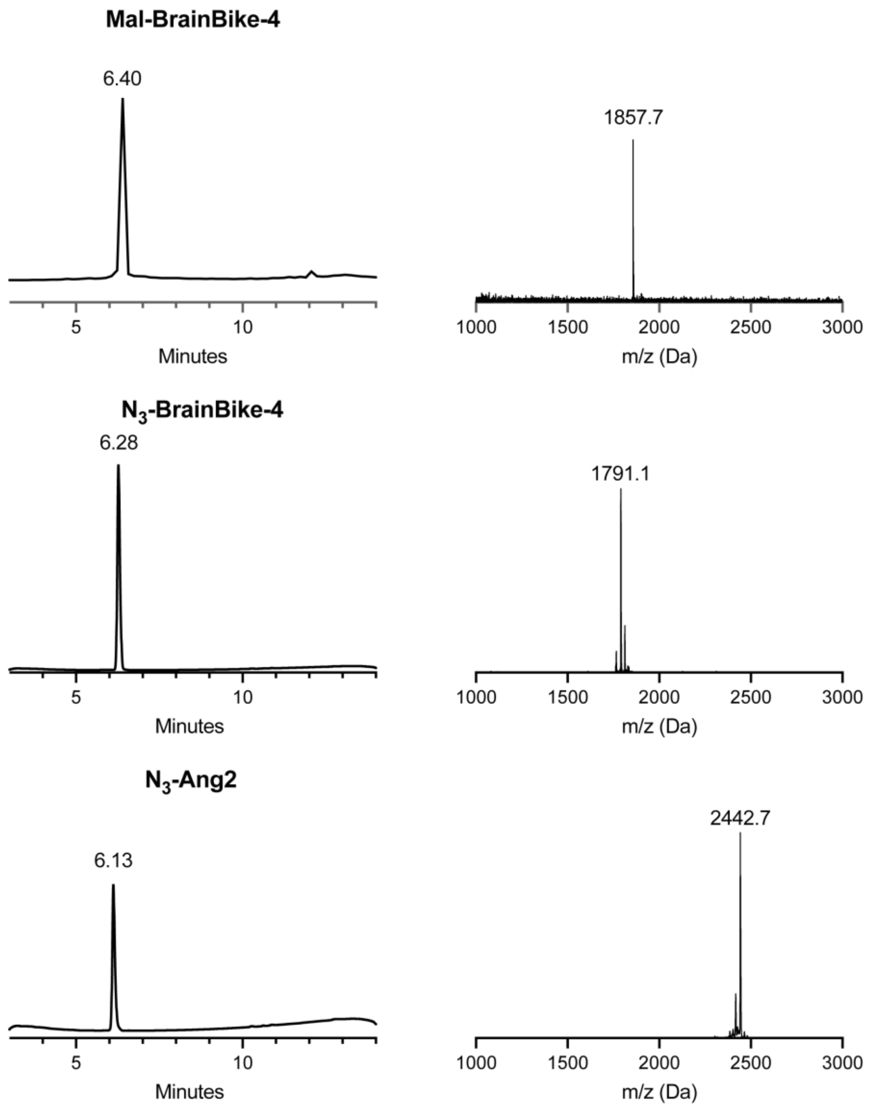

Figure S1 Continued.

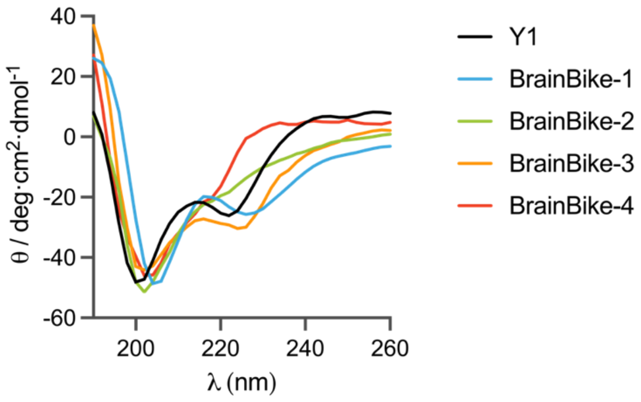

Figure S2 Circular dichroism spectra of peptides.

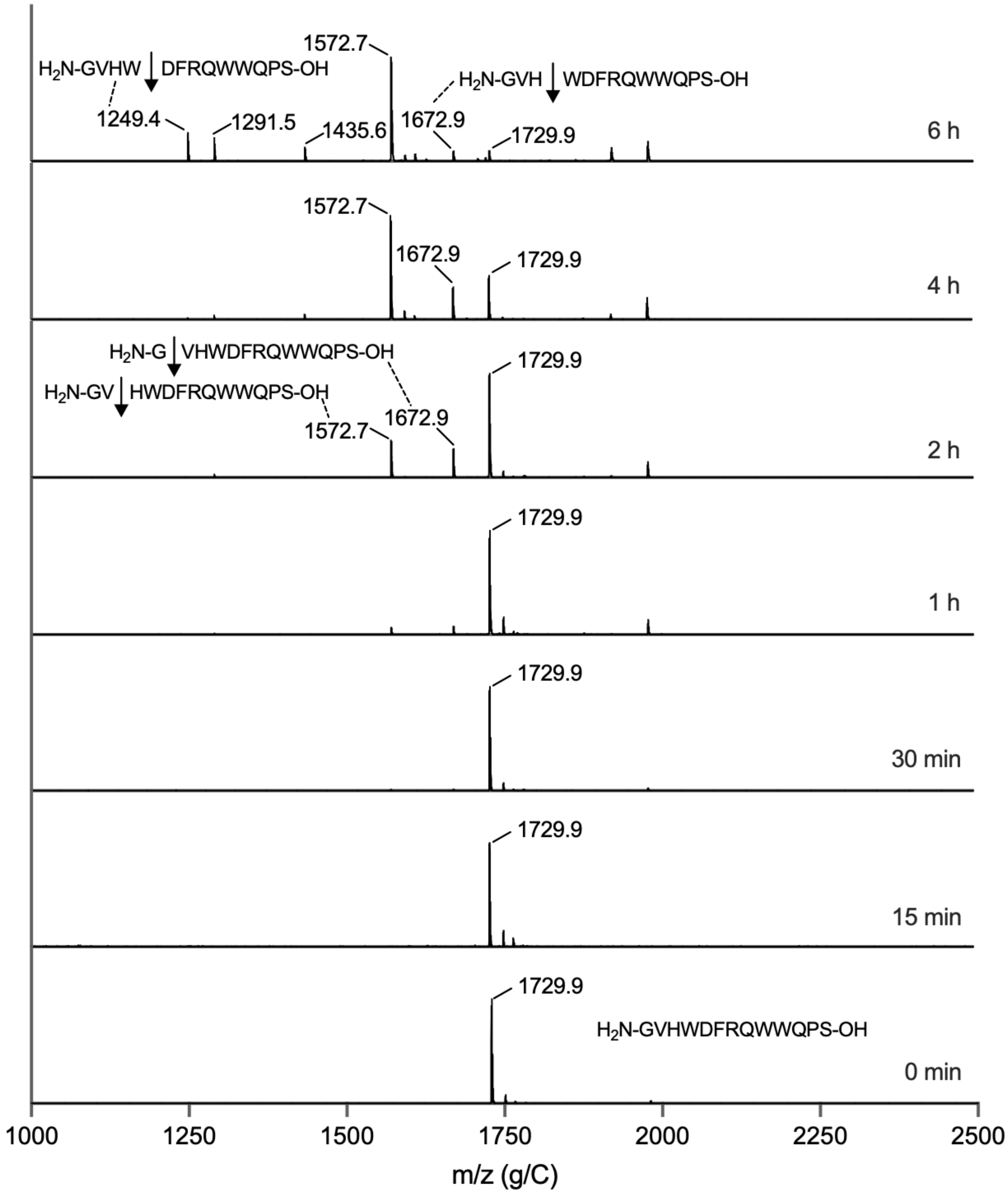

Figure S3 Mass spectra of GY1 peptide incubated in human serum at different time points.

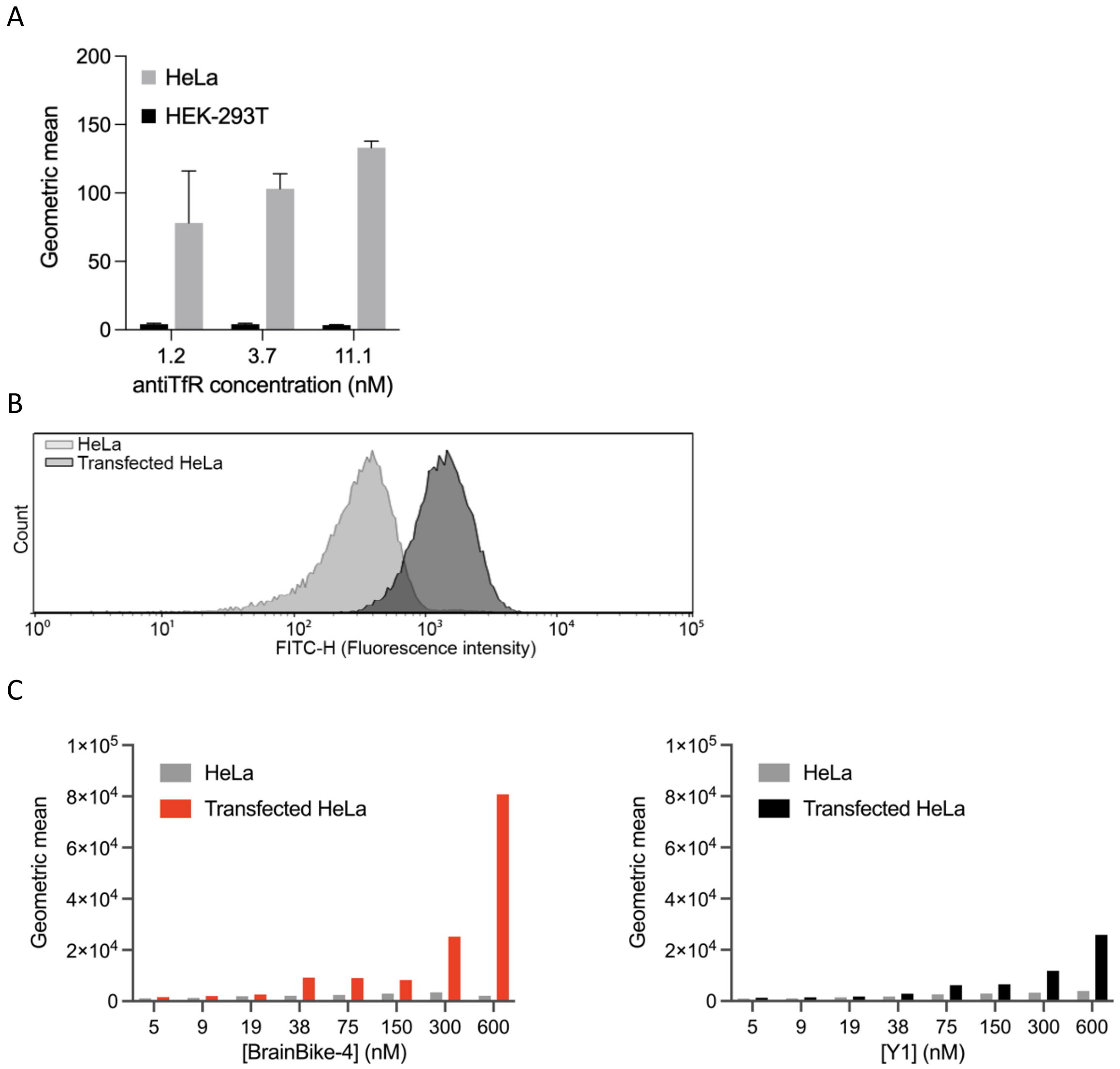

Figure S4 TfR1 expression in cells used for binding experiments. (A) TfR1 expression in HeLa and HEK-293T cell lines as assessed with a commercial anti-TfR1 antibody and flow cytometry. (B) TfR1 expression in HeLa transfected or not transfected with the plasmid encoding for TfR1-GFP, as assessed with a commercial anti-TfR1 antibody and flow cytometry. (C) Binding experiment of sCy5-BrainBike-4 (left) and Y1 (right) peptides to HeLa cells transfected (dark shade) or untransfected (light shade) with TfR1.

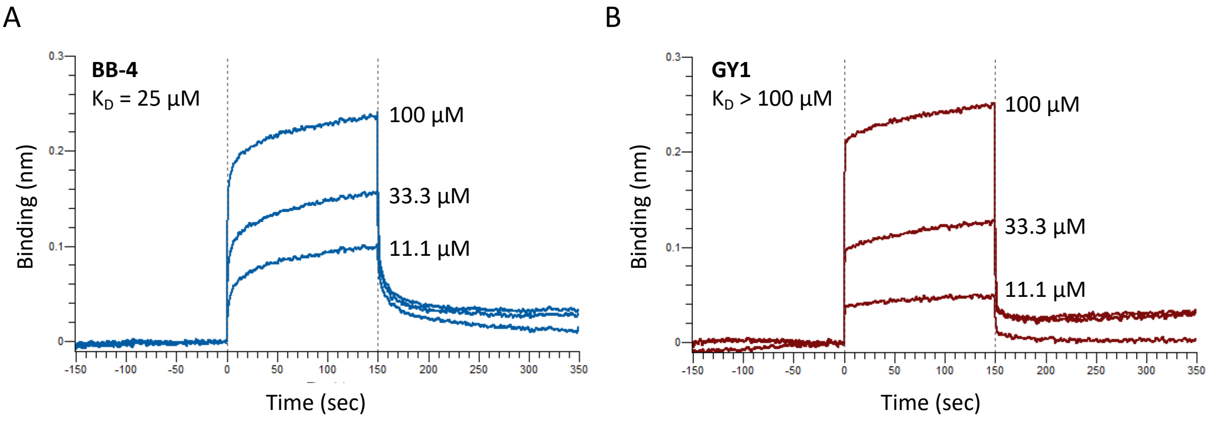

Figure S5 Binding of BrainBike-4 (A) and GY1 (B) peptides with human TfR immobilized on SA biosensors on Octet R2.

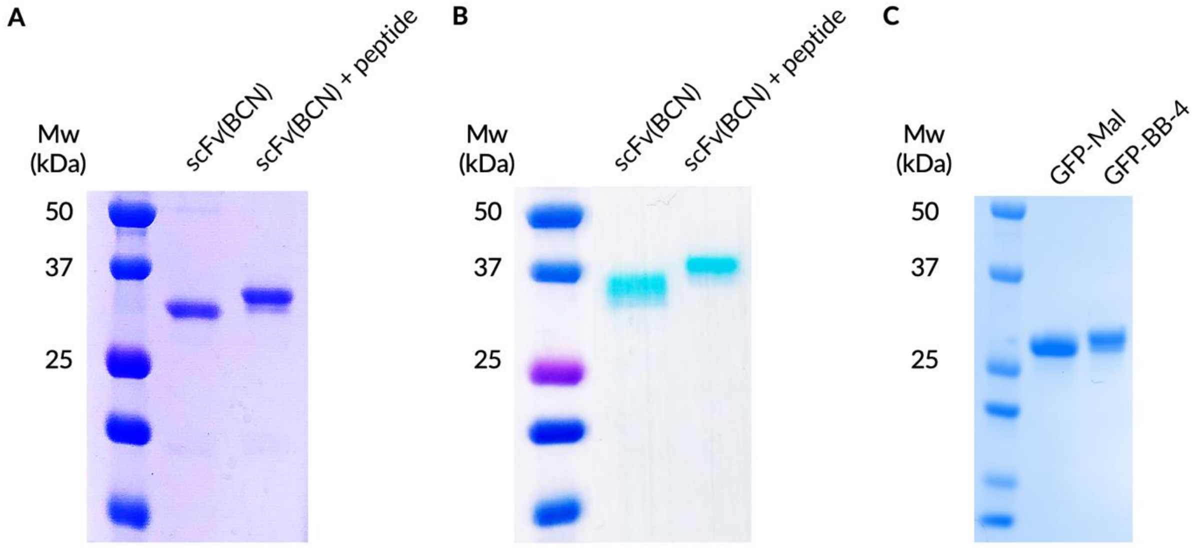

Figure S6 SDS-PAGE characterization of antibody conjugates with the peptide shuttle non-modified (A) and modified with sulfo-cyanine-5 (B). (C) SDS-PAGE characterization of GFP protein conjugation with the peptide shuttle.

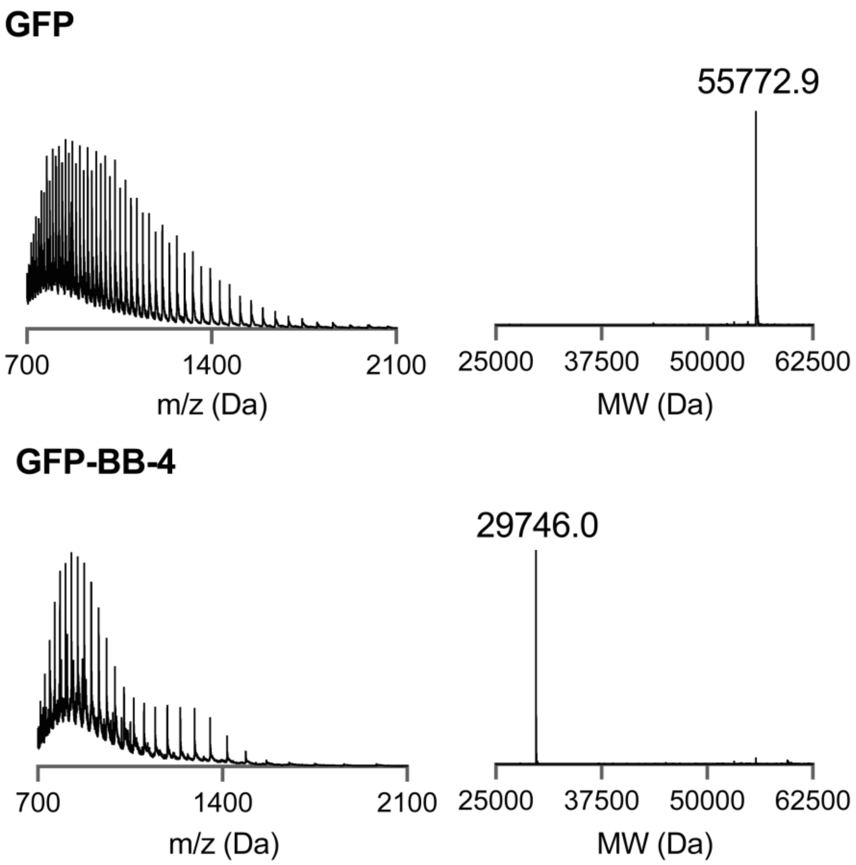

Figure S7 Characterization of GFP conjugate mass. Raw data (left) and deconvoluted mass spectra (right).

scFv

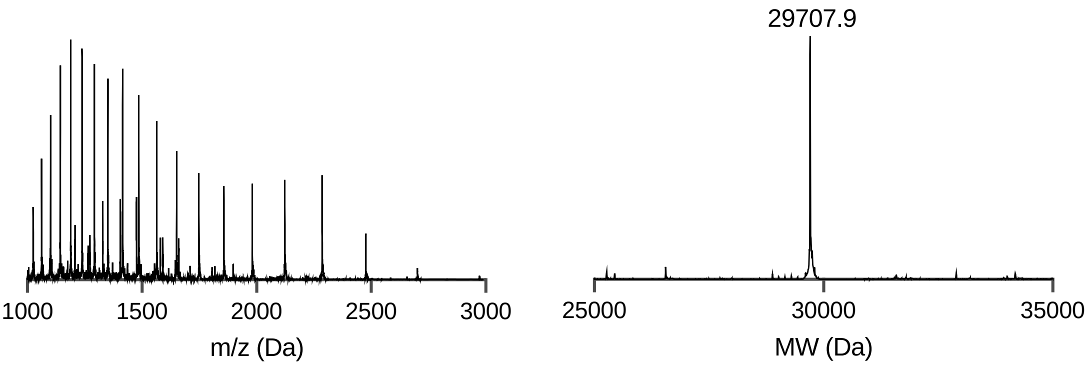

scFv(BCN)

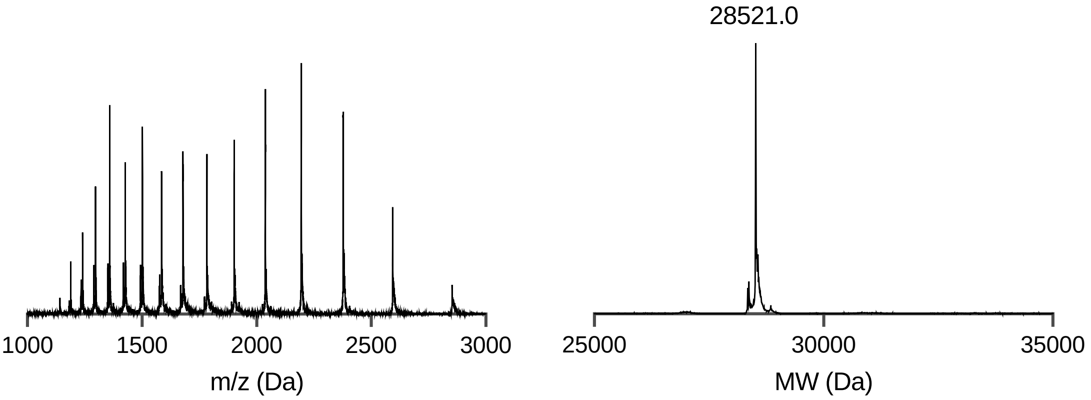

scFv-BB-4

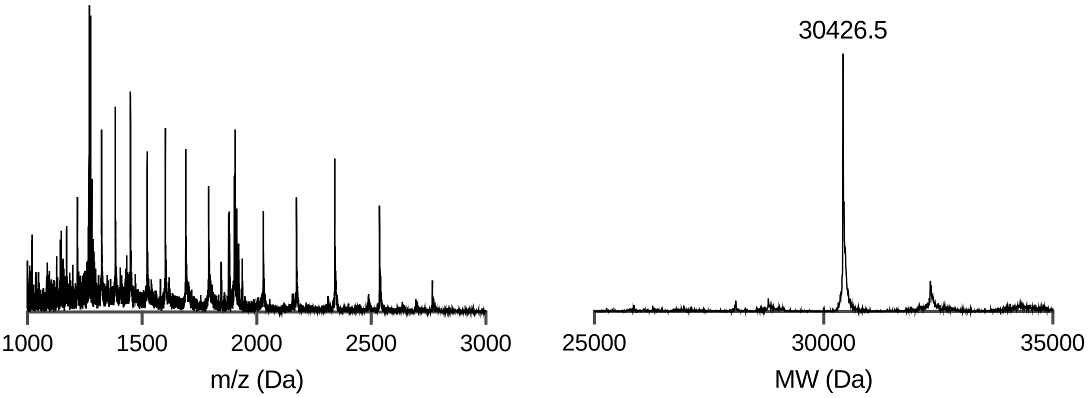

Cy5-scFv(BCN)

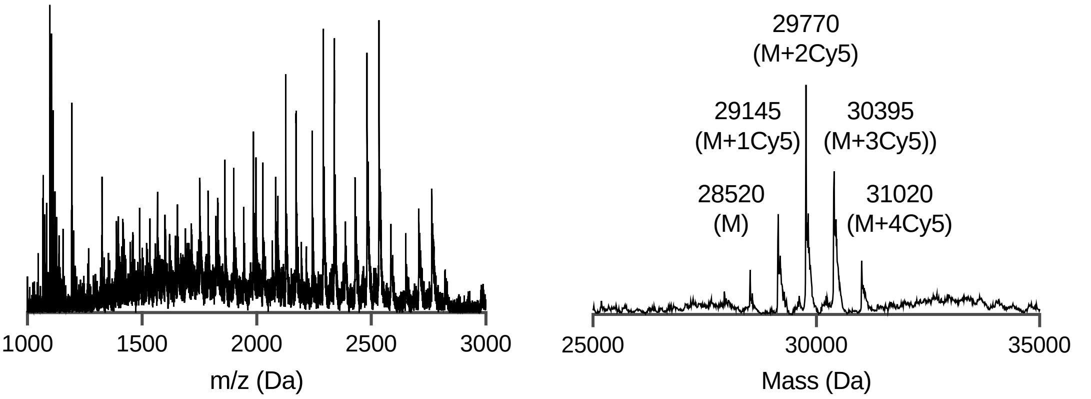

Figure S8 Characterization of antibody conjugate mass. Raw data (left) and deconvoluted mass spectra (right).
